# Modelling the resilience of seagrass communities exposed to pulsed freshwater discharges: A seascape approach

**DOI:** 10.1371/journal.pone.0229147

**Published:** 2020-02-21

**Authors:** Clinton Stipek, Rolando Santos, Elizabeth Babcock, Diego Lirman

**Affiliations:** 1 Marine Biology and Ecology Department, Rosenstiel School of Marine and Atmospheric Science, University of Miami, Miami, Florida, United States of America; 2 College of Arts, Sciences and Education, Florida International University, Miami, Florida, United States of America; Università della Calabria, ITALY

## Abstract

Submerged aquatic vegetation (SAV) communities display complex patch dynamics at seascape scales that are presently poorly understood as most studies of disturbance on SAV habitats have focused on changes in biomass at small, quadrat-level scales. In this study, analyses of remote sensing imagery and population modelling were applied to understand SAV patch dynamics and forecast the fate of these important communities in Biscayne Bay, Miami, Florida, US. We evaluated how the proximity of freshwater canals influences seagrass-dominated SAV patch dynamics and, in turn, how patch-size structure influences the stability of seagrass seascapes under different salinity scenarios. Seagrass fragmentation rates were higher in sites adjacent to freshwater canals compared to sites distant from the influences of freshwater deliveries. Furthermore, we documented a clear trend in patch mortality rates with respect to patch size, with the smallest patches (50 m^2^) undergoing 57% annual mortality on average. The combination of higher fragmentation rates and the higher mortality of smaller seagrass patches in habitats exposed to pulses of low salinity raises concern for the long-term persistence of seagrass meadows in nearshore urban habitats of Biscayne Bay that are presently targets of Everglades restoration. Our model scenarios that simulated high fragmentation rates resulted in SAV population collapses, regardless of SAV recruitment rates. The combined remote sensing and population modelling approach used here provides evaluation and predictive tools that can be used by managers to track seagrass status and stress-response at seascape levels not available previously for the seagrasses of South Florida.

## Introduction

Submerged aquatic vegetation (SAV) assemblages composed of seagrasses and macroalgae create productive ecosystems in shallow coastal environments around the world [1, 2]. These ecosystems provide a wide range of essential ecological and economic services valued at US $3.8 trillion per year [3, 4]. While serving as habitat to species such as green sea turtles and manatees, seagrass meadows also provide increasingly valuable ecosystem services such as carbon sequestration, coastal sedimentation stabilization, and improvement of water clarity [5, 6]. Furthermore, SAV facilitate trophic transfers to nearby habitats, such as marshes, mangroves, and coral reefs [7]. Seagrasses also provide the essential nursery habitat for fisheries species such as snappers, groupers, shrimp, and queen conch [8, 9].

Between 1980 and 2006, seagrasses have been disappearing at a rate of 110 km^2^ per year globally [10]. Seagrass declines have been magnified near populated coastlines due to coastal development [11, 12]. One example of coastal and watershed modifications impacting seagrass communities can be found in Florida Bay, Florida, US, from 1987–1990 and again in 2015, where mass mortality of the seagrass *Thalassia testudinum* resultedin the loss of > 4000 hectares of dense seagrass beds [13, 14]. Florida Bay is a shallow lagoon located downstream of the Florida Everglades, a watershed that has been drastically modified due to the installation of a water management canal system that has caused a reduction in the amount of freshwater reaching the bay [13]. In Florida Bay, seasonal periods of hypersalinity have been linked directly to the mass mortality of seagrasses [14].

Freshwater inputs and salinity patterns are also key drivers of seagrass abundance and distribution in Biscayne Bay, Florida. Biscayne Bay is a shallow coastal lagoon highly influenced by the quantity and timing of freshwater deliveries [15, 16]. From the early 1900s-1960s, canals were built for the drainage of agricultural and urban lands and flood prevention. While the historic salinity patterns were dominated by the slow discharge of fresh water through overland flows and groundwater, fresh water is presently primarily discharged into littoral habitats through pulsed releases from canals. This creates environments near canals that experience drastic drops in salinity (reaching 0 in some instances) over a matter of hours. These changes in the salinity regime have been linked to changes in the abundance and distribution of seagrasses and associated fauna [16–18]. In response to these significant impacts and the changes to the regional hydrology, the Comprehensive Everglades Restoration Plan (CERP) is presently being implemented to improve the quality and quantity of fresh water delivered into the coastal bays of South Florida. To document and predict the impacts of CERP, there is a pressing need to develop models and indicators that evaluate status and trends of key ecosystem indicators like seagrasses at multiple spatial and temporal scales.

Historically, the impacts of human and natural disturbances on seagrass meadows have been commonly characterized at small, quadrat-level scales, with limited attention paid to the influence of the disturbance on seagrass seascape dynamics [19, 20]. With the documentation of widespread declines and reports of localized mass seagrass mortality, there is an increasing need to evaluate response patterns at scales beyond the quadrat level [21]. Seagrass patches vary widely in size from < 1 m^2^ to hectares of continuous seagrass cover. Because of their clear boundaries (seagrass patches are commonly surrounded by sediments or rubble), seagrass patches are ideal candidates for studies of patch dynamics at seascape spatial scales.

Two previous studies in Biscayne Bay demonstrated that seagrass seascapes adjacent to freshwater canals were more fragmented than similar seascapes distant from canals, and experienced wide fluctuations in cover and fragmentation rates over time [22, 23]. Still, these prior studies did not directly address nor quantified detailed patch dynamics that are important to identify mechanisms of fragmentation and help forecast seascape stability patterns under different salinity scenarios. Patch dynamics such as changes in patch size structure as well as patch mortality and growth rate are known to influence the stability and local extinction probability of habitats composed of terrestrial and marine plant species [22, 24–26]. Thus, this study examined the long-term dynamics of seagrass/SAV patches in Biscayne Bay in association with freshwater deliveries by analyzing the historical response of SAV/seagrass patches of different sizes within distinct salinity environments. We hypothesized that the rates of fragmentation and thus the long-term dynamics of patch-size structure, would be influenced by the discharge of fresh water from canals, with areas closer to canals having seascapes with higher fragmentation rates and patch-size structures dominated by smaller patches that can compromise the long-term persistence of SAV/seagrass habitats.

## Materials and methods

### Study design

The hydrology and salinity patterns of western Biscayne Bay are influenced by the location and flow rates of drainage canals. Areas near canals can exhibit extreme oscillations in salinity levels and this pattern is heightened during the wet season (July to October) when freshwater is released in pulses to drain the Florida Everglades and upstream urban and agricultural areas [22].

**Fig 1:**
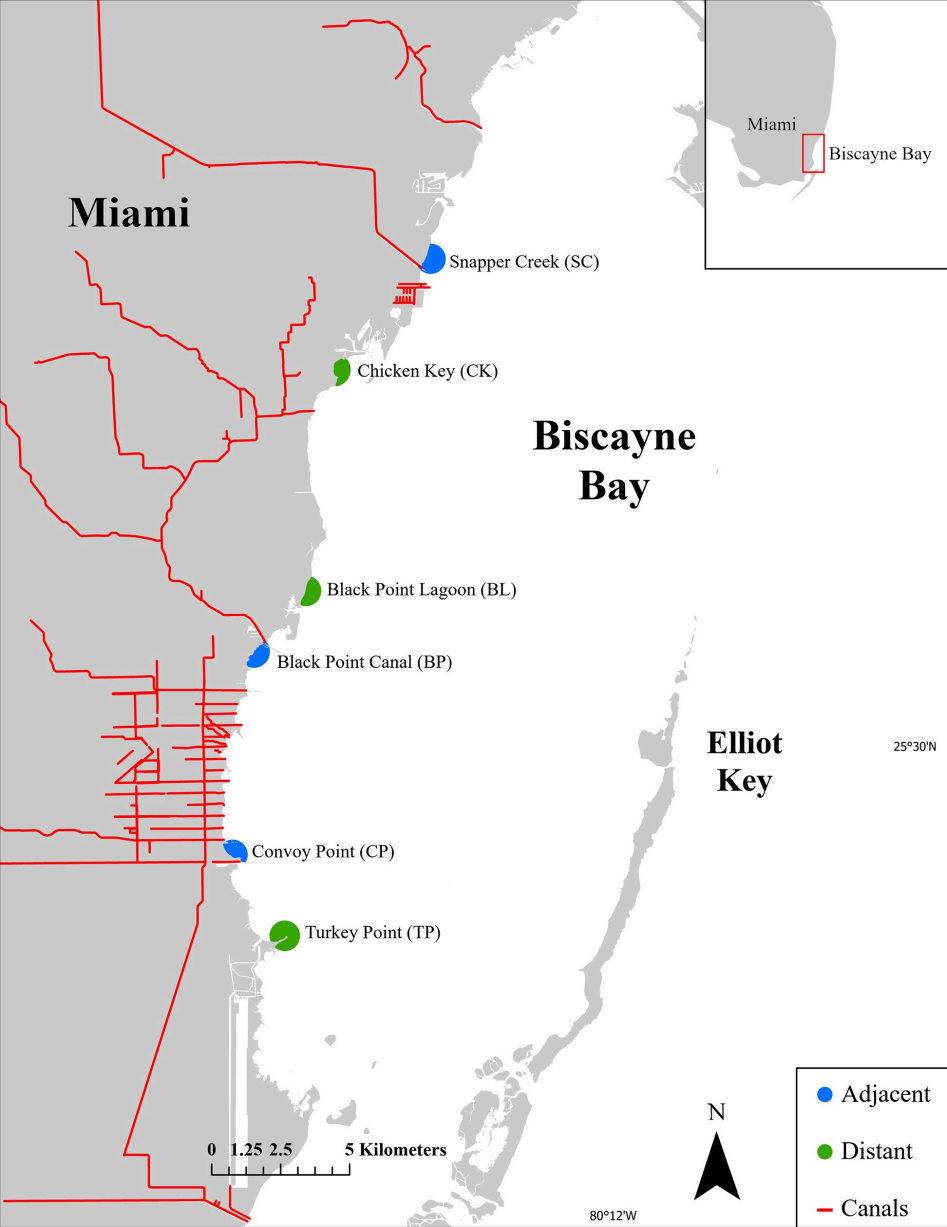
Location of study sites for this project. **The sites in green outline are considered “distant” to canals, with a mean distance of 2.8 (± 0.9) km from canals. Sites in blue are considered “adjacent” to canals, with a mean distance of 0.5 (± 0.1) km the nearest canals**. SC: Snapper Creek, CK: Chicken Key, BL: Black Point Lagoon, BP: Black Point Canal, CP: Convoy Point, TP: Turkey Point. Red lines show the location of the freshwater canals.

Six sites were selected along the western shoreline of Biscayne Bay where the impacts of CERP are concentrated. The sites considered ‘adjacent’ (Snapper Creek, Black Point Canal, Convoy Point) were in proximity to canals with the highest freshwater discharge volumes within Biscayne Bay (Fig 1). Paired ‘distant’ (Chicken Key, Black Point Lagoon, Turkey Point) sites were randomly selected at distances > 1 km^2^ from a canal (Fig 1). Each site encompassed a 500-m buffer around a location selected along the shoreline as described by Santos et al. [23]. Historical aerial photos of these six sites were assessed over nine periods, 6–13 years apart from 1938–2009 based on the availability and quality of aerial imagery. Salinity data collected using YSI instruments deployed in the vicinity of each site from 2010–2015 showed that sites adjacent to canals had lower average salinity (24.4 g/L) compared to sites distant from canals (29.3 g/L). The research described in this study was conducted through remote sensing and GIS and thus did not require any scientific permits.

### Spatial analyses

Seagrass maps were created using black-and-white aerial photographs obtained from local government agencies. These images were processed to standardize their resolution, optical properties, and sampling area. Images were geo-rectified using a United States Geological Survey topographic map as a spatial reference (for mapping details see [23]). Seagrass maps were created by hand-digitizing individual seagrass patches at 1:2500 scale. For the purpose of this study, individual seagrass patches were classified into the following five size classes: 1) Size 1 (<50 m^2^); 2) Size 2 (50–100 m^2^); 3) Size 3 (>100–500 m^2^); 4) Size 4 (>500–2000 m^2^); and 5) Size 5 (>2000 m^2^).

### Patch dynamics

**Fig 2:**
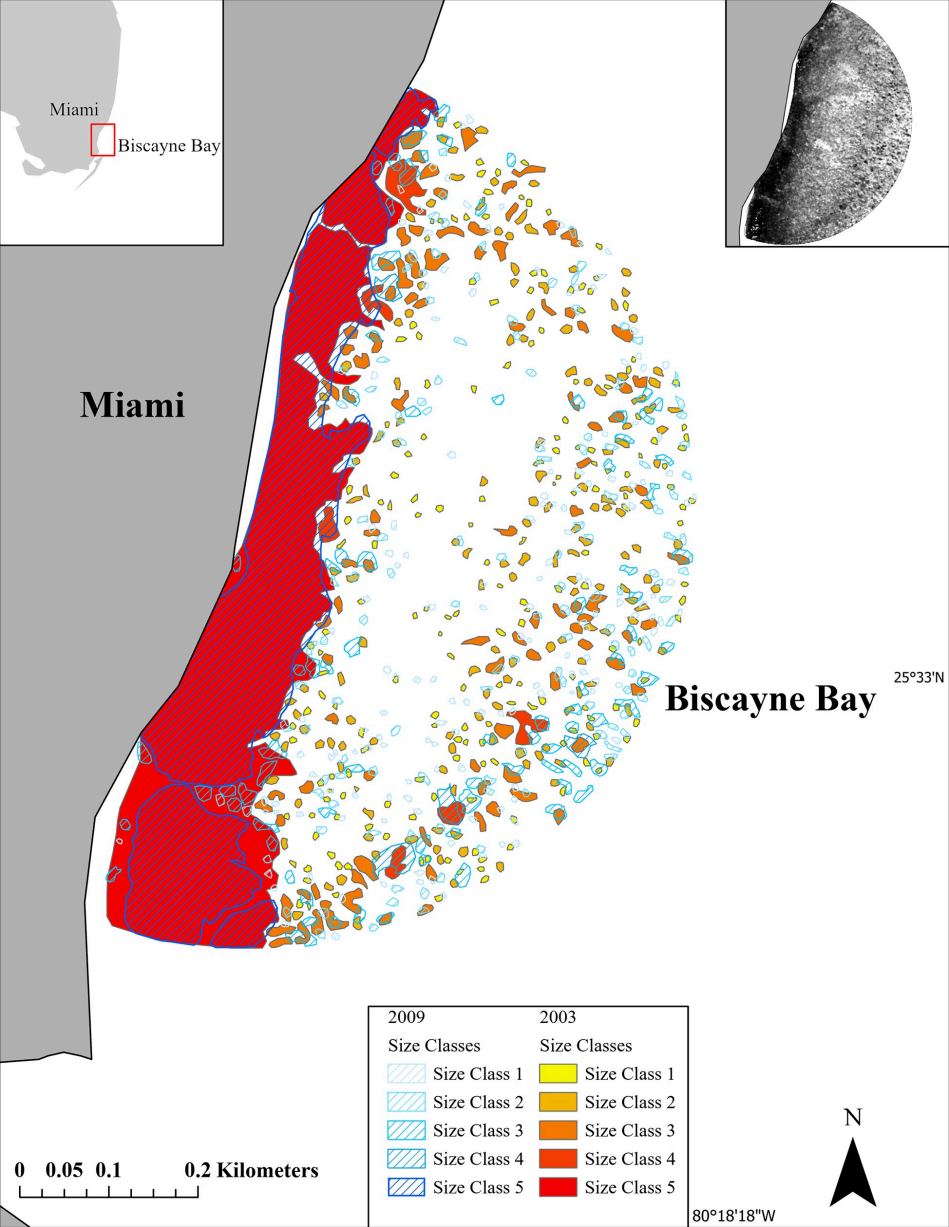
SAV patches tracked at the Black Point Lagoon sites between 2003–2009. **The 2009 patches appear overlaid on top of 2003 to visualize changes over time.** Insert in the top right is an example of an aerial image used to track SAV patches in this study.

Population models based on size rather than age are particularly useful in describing the dynamics of plants and clonal invertebrates [27, 28]. In this study, five seagrass patches from each of the size class were randomly selected for each site at every time interval, as this was the highest number of patches, on average, that would provide equal representation across all five size categories consistently. To evaluate the fate of each patch between time steps, the GIS map for the end of a time interval (t_1_) was superimposed on top of the map for the beginning of the time interval (t_0_) (Fig 2). The fate of each of the five selected patches/polygons per size class was recorded as growth, shrinkage, fragmentation, merging, or mortality using the following rules:

1. For a fate to be classified as “**growth**”, a patch identified in t_1_ had to overlap with the original t_0_ patch and show an increase in area between t_0_-t_1_.
2. **Shrinkage** was recorded in the opposite way; where the t_1_ patch had to be the only patch in contact with the t_0_ patch and show a decrease in area between t_0_-t_1_.
3. **Fragmentation** was recorded if the original patch in t_0_ divided into more than one new patch in t_1_.
4. **Merging** was recorded if distinct t_0_ patches joined together to form a new patch in t_1_.
5. **Mortality** was recorded when the original patch disappeared between t_0_-t_1_.

The fate data were used to develop the following transition matrix:

$$N_{\left(t+1\right)}=A\cdot N_{\left(t\right)}$$

where **A** is a Leslie matrix describing the probabilities of transition between size classes and **N**_**(t)**_ is the population vector that describes the number of individuals in each size category at time t [29]. Transitions were expressed as proportions. For example, if two of the five size-1 patches identified in t_0_ grew to size 3 in t_1_, 0.4 would be recorded as growth from size 1 to size 3. If three of the size-3 patches from t_0_ shrunk to size 1 in t_1_, 0.6 would be recorded as shrinkage.

Our model accounts for recruitment of new seagrass patches into the population by adding recruits as a proportion of the existing patches in t_o_ to the first row in the matrix [29, 30]. To estimate recruitment, the patches that appeared on previously unoccupied space in t_1_ and were not in contact with any patches from t_0_ were identified and counted as recruits. The total number of recruits identified was divided by the total number of patches recorded at t_0_ to calculate the per-patch recruitment rate that was added to the first row of the Leslie matrix. If, for example, 10 new patches (recruits) were detected in t_1_, this number was divided by the total number of patches in t_o_ (e.g., 100) to provide a recruitment rate of 0.1. Recruitment rates were calculated on a yearly basis for standardization.

Merging was considered as a special case of growth into larger size categories, where the growth transition probabilities for each patch size merging were adjusted by dividing by the number of patches that merged. If, for example, one of the five (0.2) size-3 and one of the five (0.2) size-4 patches merged to form a single size-5 patch, the growth probability for size 3 to size 5 would be 0.2/2. Similarly, the growth probability for size 4 to size 5 would be 0.2/2. This increases the abundance of size-5 patches and decreases the abundance of the smaller, merging size classes proportionally to their contribution.

Fragmentation was treated as a special case of recruitment, where a larger patch can produce several smaller patches through fission. The model accounts for both the possibility that the parent patch declines in size through fragmentation (by transitioning/shrinking into a smaller size class) or remains within the same size class even after fragmentation (especially true for larger size-5 patches that can undergo fragmentation and still retain their large size). The fate of the parent patch is tracked as any other patch that can grow, shrink, or stay within the same size category. For example, if one of the size-5 patches produced 50 new size-1 patches through fission, the per-patch fragmentation rate would be 50 divided by the t_0_ population (200) to provide the fragmentation rate of 0.25 to size class 1. To compare between sites, annual rates of fragmentation were calculated.

The ‘popbio’ package in R was used for the analysis of the population dynamics and to calculate lambda (λ, eigenvalue) and the stable size-frequency distributions (eigenvectors) from the Leslie matrix [30]. The eigenvalues calculated the growth rate and various demographic parameters drawn from the projection matrix. A λ > 1 indicates the population size (i.e., number of patches) is growing while a λ < 1 indicates population size shrinkage. The lambda values obtained for the uneven time steps were converted to a yearly rate by taking the root of the lambda to the years in the time step.

### Population projections and fragmentation scenarios

The transition and population structure information collected here were used to run population projections based on Leslie matrices built under different recruitment and fragmentation scenarios to evaluate the long-term impacts of these key patch-size structure processes under different salinity environments. The function ‘pop.projection’ was applied to project the changes of the transition matrices into the future using the Leslie matrix multiplied by the respective population vector. These population vectors, built by time step and by site, were multiplied by the associated transition matrix for 17 intervals, with each interval representing 5 years. The function ‘stage.vector.plot’ was then used to visualize the results to identify when the population converged to steady state distributions [30].

Population projections were run under low and high fragmentation scenarios. The transition values used to represent high and low fragmentation conditions were determined based on the transition data collected in this study by selecting one site/time interval that displayed fragmentation rates above and one below the global averages. These scenarios were then run with low, average, and high recruitment values selected similarly. All scenarios used to evaluate the change in the structure of the eigenvector were run with equal proportions of size classes as starting conditions. The scenarios to project the change in population abundance were run with the average abundance for each size class recorded in this study for all sites and times combined as starting conditions.

The influence of patch size and salinity environment (i.e., adjacent vs. distant) on patch mortality rates was evaluated using a two-way ANOVA, where the response variable, mortality, was normalized through a logit transformation. The relationship between recruitment rates and the number of SAV patches was evaluated using linear regression.

### Caveats

One of the limitations of this study was the inconsistent seasonality of the aerial imagery. Seagrasses are known to undergo seasonal changes in biomass [18, 31]. Thus, the lack of consistent seasonal aerial imagery prevented us from accounting for differences due to seasonality. Furthermore, the resolution of the imagery was insufficient to distinguish the various macrophyte species that compose the SAV communities. This low taxonomic resolution may mask changes in community composition from euhaline (*Thalassia testudinum*, *Halimeda* spp.) to mesohaline taxa (*Halodule wrightii*, *Laurencia* spp.) [23]. Also, the resolution of the imagery does not allow for visualization of biomass thinning, which can be a precursor to fragmentation. The use of aerial images may have also resulted in an underestimation of recruitment rates as very small patches were impossible to detect due to the spatial resolution of the data. A more accurate determination of patch formation and recruitment that is based on field surveys would be needed to provide a better understanding of recruitment rates and their influence on seagrass patch dynamics in the future. Lastly, while the salinity data were not available for the > 70 years of SAV data available through aerial imagery, salinity patterns have been spatially consistent for the period of record (>15 years) when salinity has been tracked within Biscayne Bay [32, 33], supporting our assessment that distinct salinity environments influenced by freshwater discharges have been present in Biscayne Bay for decades.

## Results

### Patch mortality rates

**Fig 3:**
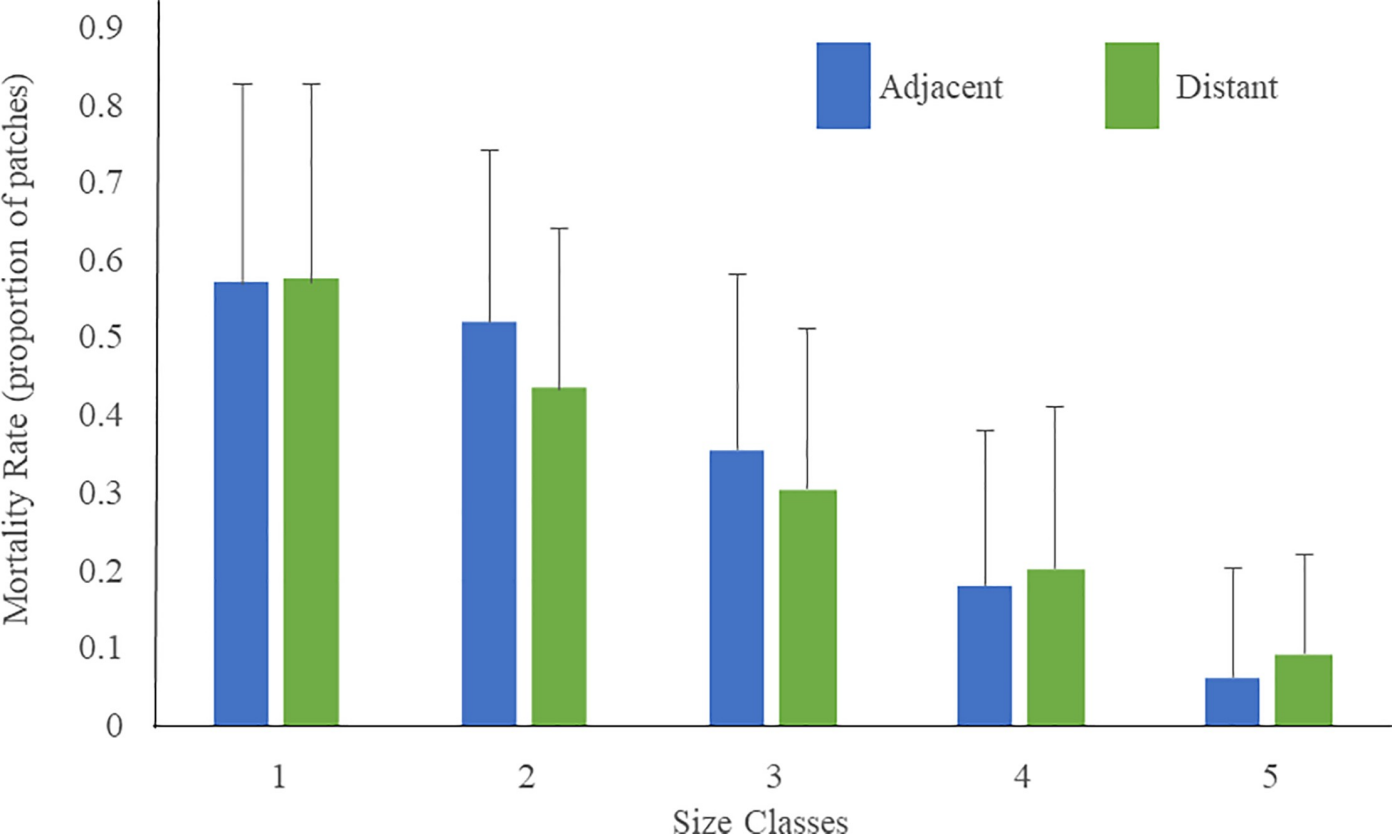
Average mortality rates in relation to patch size documented at the Black Point Lagoon site. **Values represent average (± SD) annual mortality rates for all time periods combined.** n = 27 (3 sites x 9 time periods).

Seagrass patch size played a significant role on mortality rates, with the smallest size classes (1 and 2) having significantly higher mortality than the largest size classes (4 and 5) (ANOVA, Tukey test, p<0.05; Fig 3). No significant effects of salinity environment on mortality were found (p>0.05), and no significant interactions between the two factors were documented (p>0.05).

### Recruitment

On average, 23 (SD = ± 25) new seagrass patches per time period were observed within the 500-m radius study sites when all data (i.e., times and sites) were combined. The average annual recruitment rate (i.e., number of recruits at t_1_ /number of patches at t_0/_ time) was 2.7 (SD = ± 3.7) for all times and sites combined. The number of recruits showed a significant positive relationship with the total number of patches in each site (linear regression, p<0.05). No significant patterns in the number of recruits per site were detected based on total SAV coverage, the abundance of size-5 patches, or lambda values (linear regression, p>0.05 for all 3 factors). Finally, no significant differences in the numbers of recruits were found between adjacent and distant sites (t test, p>0.05, all years combined).

### Fragmentation rates

Sites adjacent to canals had a mean annual rate of 6.2 patches yr^-1^ (SD = ±4.6) created through fragmentation compared to distant sites (4.0 patches yr^-1^, SD = ±3.4) when all time periods were combined (Table 1). While fragmentation was 1.5 times higher in sites closer to canals, this difference in mean fragmentation rates was not significantly different between salinity environments (t-test, p = 0.09). For adjacent sites BP, CP, and SC, the average number of fragments produced per year were 6.4, 6.6, and 5.7, respectively. For distant sites BL, CK, and TP, the average number of fragments produced per year were 5.7, 2.7, and 3.4, respectively. No significant relationship was found between fragmentation rates and time for either the adjacent (linear regression, p>0.05) or distant sites (linear regression, p>0.05).

**Table 1:** Fragmentation rates (N patches created per year) of adjacent and distant sites over all time steps.

Time Step	Adjacent	Distant
**1938–1944**	5.7	3.9
**1944–1950**	8.5	3.5
**1950–1963**	2.2	1.7
**1963–1973**	7.4	2.8
**1973–1985**	5.2	2.3
**1985–1991**	4.1	8.9
**1991–2003**	11	5.9
**2003–2009**	5.7	2.7
**Average**	6.2	4

### Patch dynamics

Lambda values varied spatially and temporally, without any clear patterns (Table 2). In fact, no temporal patterns in the yearly *λ* values were recorded (linear regression, p>0.05) for all sites combined. For adjacent sites, two of the three sites showed an average of *λ* ≥ 1 over all time periods, representing a growing number of seagrass patches. Only one of the distant sites (BL) displayed an average *λ* >1 over all time periods. While BL had the highest fragmentation rates of any of the distant sites, the fragments produced were of a larger size class, on average, than at the adjacent sites. The largest average *λ* values across all sites was documented for the 1963–1973 time step (*λ* = 1.03). The lowest average *λ* values across all sites were documented for the 1973–1985 and 2003–2009 time intervals (*λ* = 0.97). The highest average *λ* values for the adjacent sites was 1.08 (SD± = 0.09) from 1963–1973. The highest average *λ* for the distant sites was 1.07 (SD± = 0.08), recorded during the 1985–1991 time step. The lowest *λ* recorded for adjacent sites was 0.95, displayed in 2003–2009. The lowest *λ* for distant sites was 0.92 recorded from 1950–1963. No significant differences in average *λ* were found between distant (mean *λ* = 0.99 (±0.07)) and adjacent sites (mean *λ* = 1.0 (±0.07)) (t-test, p>0.05).

**Table 2:** Yearly lambda (*λ*) values for all sites and time steps.

	Sites	1938–1944	1944–1950	1950–1963	1963–1973	1973–1985	1985–1991	1991- 2003	2003–2009	Average
**ADJ**	SC	0.95	N/A	1.03	1.01	0.98	0.96	0.96	0.95	0.97
**DIS**	CK	1.03	1.03	0.93	0.95	1.03	1.00	0.96	0.93	0.98
**DIS**	BL	1.02	1.00	0.91	1.02	0.92	1.16	0.98	1.15	1.02
**ADJ**	BP	1.00	0.93	0.97	1.19	0.94	.96	1.14	0.97	1.01
**ADJ**	CP	1.02	1.05	1.05	1.04	0.96	0.96	1.06	0.98	1.01
**DIS**	TP	0.92	1.03	N/A	0.96	0.97	1.06	0.96	0.92	0.97
	Average	0.99	1.00	0.98	1.03	0.97	1.02	1.01	0.97	
	Std. Dev.	0.044	0.047	0.061	0.086	0.04	0.077	0.076	0.092	

ADJ: Adjacent to canals, DIS: Distant from canals.

The stable population proportions determined by the eigenvectors showed that size-3 patches composed the highest, on average, proportion of the population at 0.38 when all sites are combined (Table 3). Size 1 had the next highest proportion at 0.23, with size 2 composing 0.21 of the population. Sizes 4 and 5 had the lowest proportions with values of 0.12 and 0.05 respectively.

**Table 3:** Proportion of the population (i.e., eigenvectors) composed by each seagrass patch size class over time averaged across the six sites.

Time Step	Size 1	Size 2	Size 3	Size 4	Size 5
**1938–1944**	0.11	0.20	0.45	0.17	0.06
**1944–1953**	0.27	0.20	0.37	0.12	0.05
**1953–1963**	0.23	0.20	0.42	0.10	0.07
**1963–1973**	0.22	0.19	0.42	0.10	0.07
**1973–1985**	0.16	0.24	0.36	0.17	0.07
**1985–1991**	0.31	0.23	0.33	0.09	0.04
**1991–2003**	0.31	0.21	0.38	0.07	0.03
**2003–2008**	0.25	0.23	0.32	0.17	0.04
Average (±STD)	0.23 (0.07)	0.21 (0.02)	0.38 (0.05)	0.12 (0.04)	0.05 (0.02)

### Population projections and fragmentation scenarios

All scenarios were run over 17, 5-year intervals. Low fragmentation and high fragmentation scenarios used a fragmentation rate of 0.8 and 10.2 patches created per existing patch, respectively. Low recruitment scenarios used a recruitment rate of 0.007 patches created per existing patch, average recruitment was 0.15 patches created per existing patch, and high recruitment was 0.6 patches created per existing patch. The stable size distribution for the high fragmentation/low recruitment scenario was 0.39, 0.43, 0.11, 0.01, and 0.06 for the respective size classes. The stable size distribution for the low fragmentation/low recruitment scenario was 0.06, 0.32, 0.41, 0.11, and 0.10 for the respective size classes. The stable size distribution for the high fragmentation/average recruitment scenario was 0.55, 0.37, 0.01, 0.02, and 0.05 for the respective size classes. The stable size distribution for the low fragmentation/average recruitment site was 0.21, 0.08, 0.33, 0.20, and 0.17 for the respective size classes. The stable size distribution for the high fragmentation/high recruitment scenario was 0.66, 0.29, 0.01, 0.01, and 0.03 for the respective size classes. The stable size distribution for the low fragmentation/high recruitment scenario was 0.27, 0.04, 0.32, 0.18, and 0.19 for the respective size classes.

**Fig 4:**
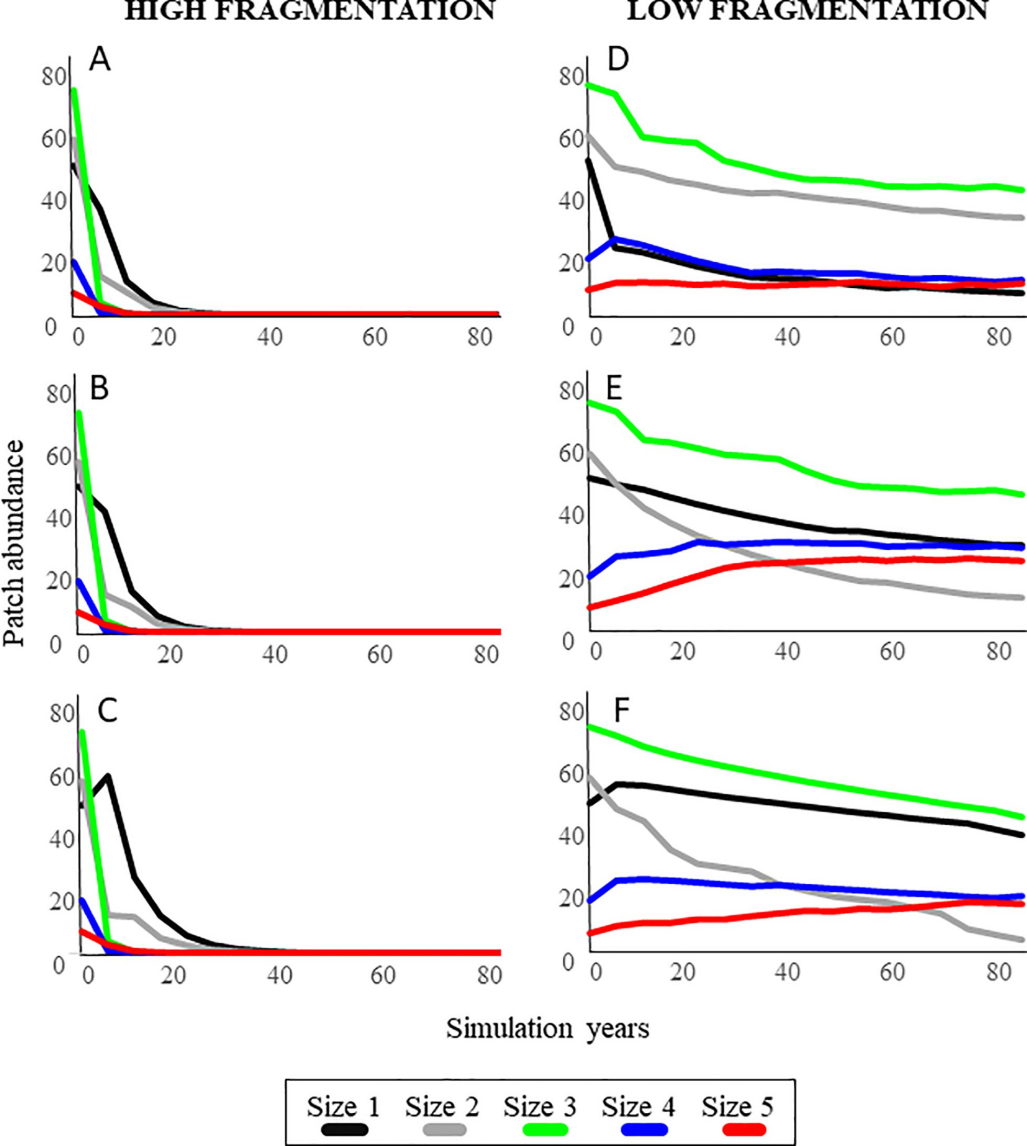
SAV patch abundance patterns over time simulated under high fragmentation (left panels) and low fragmentation (right panels) for (A, D) low recruitment, (B, E) average recruitment, and (C, F) high recruitment scenarios.

Scenarios were run with an initial population vector composed of 200 patches divided into 48 (size 1), 56 (size 2), 72 (size 3), 17 (size 4), and 7 (size 5) patches based on the average conditions recorded in this study. SAV population abundance declined to near zero in the high fragmentation scenarios regardless of recruitment rates (Fig 4). In contrast, seagrass populations persisted under low fragmentation scenarios and abundances for the larger size classes (4 and 5) increased as recruitment increases (Fig 4).

## Discussion

Seagrasses around the world have experienced multiple disturbances that have resulted in accelerating rates of loss of these important coastal ecosystems [10, 34]. Within this worldwide context of degradation, nearshore habitats of Biscayne Bay have shown remarkable resilience, having experienced, on average, only < 4% declines in SAV cover over 70 years [23]. This limited decline is also noteworthy considering that the nearshore habitats of Biscayne Bay have been significantly modified over the past 5–6 decades by the construction of a canal system that has not only reduced the overall amount of fresh water reaching Biscayne Bay but also created large gradients in salinity [16, 35]. Our historical seascape and patch dynamic analyses shows that seagrass stability has not been greatly affected by these changes and that seagrass meadows appear to be both resistant and resilient to the modified salinity patterns. The fluctuation in lambda values documented, showing alternating periods of population decline with periods of population growth due to variable disturbance regimes, are further evidence of SAV resilience patterns in Biscayne Bay.

While the limited historical loss of seagrasses cover documented here and previously by Santos et al. [23] is a positive result compared to the multiple reports of declines elsewhere, our patch-based seagrass population model developed based on the historical imagery showed that fluctuating salinity found near canals does have a negative impact through the fragmentation of seagrass meadows. Thus, even if seagrass habitats in Biscayne Bay have retained areal coverage, they are showing signs of salinity-driven fragmentation. Reports from other systems have shown that fragmented habitats are more susceptible to further disturbance and that they can decline rapidly [36–38]. Our simulation scenarios suggest that, under persistent high rates of fragmentation, seagrass populations may fall below a resistance threshold and decline rapidly thereafter. While recruitment of new seagrass patches through sexual or asexual reproduction may, to some extent, mitigate the impacts of high fragmentation, here we found that populations under simulated continuous high fragmentation scenarios can disappear within 50 years.

This study is the first to quantify the patch dynamics of seagrass communities in South Florida and our seascape approach has revealed key aspects of how seagrass populations respond to differing environmental conditions. The mortality rate of seagrass patches was significantly affected by patch size, with the mortality rate of the smallest patches (57%) being an order of magnitude higher than that of the largest patches (< 5%). Similar size-based patterns of mortality were documented for corals and sponges, clonal/colonial taxa that also showed higher mortality of the smaller size classes [28, 39]. Smaller seagrass patches have been shown in other studies to have higher susceptibility to physical disturbances [25, 38]. The high mortality rate of smaller patches could be due to their lower biomass-to-perimeter ratio that may limit their anchoring capabilities as well as expose them to higher erosion rates along the patch perimeter [23]. Larger patches may have a more extensive root system with higher storage of below-ground biomass that can help increase their resilience and lead to lower mortality rates.

Considering the large difference in mortality rates among patch sizes, any shift in population structure that reduces mean patch size (e.g., fragmentation) would reduce the resilience of seagrass populations. This pattern was captured as output of our simulated scenarios that showed rapid population declines under high fragmentation scenarios. In Biscayne Bay, 94% of the seagrass patches created by fragmentation were produced by the two largest size classes. The stability in the cover of the seagrass meadows recorded over the 70-year period of record by Santos et al. [23], even when fragmentation rates were high in some time periods, is likely due to the fact that the larger patches were able to fragment and still remain within the large size classes that provide a size refuge against mortality. Continued fragmentation would, eventually, lead to a reduction in the abundance of these large and stable patch sizes, resulting in the population declines observed in our simulations. The persistence of the simulated seagrass populations was directly related to the fragmentation rates that affected the proportion and size distribution of patches. Under high fragmentation scenarios, the abundance of larger patches declined quickly. Without the source of new small patches through fragmentation as these larger patches decline, the populations disappeared within 50 years, regardless of recruitment rates. In contrast, under low fragmentation scenarios, the abundance of larger patches remains stable or even increases over time, leading to the persistence of the population. Thus, our model showed that, along with fragmentation rates, the abundance of larger size classes plays a major role in stabilizing the seagrass community. Of special concern would be scenarios in which both an increase in fragmentation and a decrease in percent cover co-occur, as these declines can cascade into reductions of macrofaunal species richness, biomass, diversity, composition, and habitat availability [23, 40].

The population model developed here provides a tool that can be used by managers for the early detection of undesired impacts of changes in water quality on susceptible coastal resources. This seascape approach, combined with the more focused monitoring of seagrass biomass, can inform the adaptive management framework in place for the Comprehensive Everglades Restoration Plan about areas of concern and the need to modify freshwater delivery plans in combination with active restoration to prevent future seagrass losses. Using this approach managers can identify conditions under which seagrass communities within Biscayne Bay are at higher risk than others. For example, if a community that is adjacent to a canal is displaying high fragmentation and has high proportions of SAV patches within the smaller size classes, the seagrasses may be at risk of collapsing. To mitigate this risk, the fresh water should either be discharged as sheet-flow across longer shoreline sections, stopped, or shifted to a canal where the nearby SAV community is in a better condition (i.e., exhibits lower fragmentation rates).

## Supporting information

S1 File This file includes the survivorship data for each patch based on size class. (XLSX)

S2 File This file includes the abundance of patches of different sizes for the high and low fragmentation scenarios under low, average, and high patch recruitment. (XLSX)
